# Infection Dynamics and Genomic Mutations of Hepatitis E Virus in Naturally Infected Pigs on a Farrow-to-Finish Farm in Japan: A Survey from 2012 to 2021

**DOI:** 10.3390/v15071516

**Published:** 2023-07-07

**Authors:** Masaharu Takahashi, Satoshi Kunita, Tsutomu Nishizawa, Hiroshi Ohnishi, Putu Prathiwi Primadharsini, Shigeo Nagashima, Kazumoto Murata, Hiroaki Okamoto

**Affiliations:** 1 Division of Virology, Department of Infection and Immunity, Jichi Medical University School of Medicine, 3311-1 Yakushiji, Shimotsuke, Tochigi 329-0498, Japan; mtaka84@jichi.ac.jp (M.T.); tnishizawa@jichi.ac.jp (T.N.); hions.easy@gmail.com (H.O.); thiwik8@jichi.ac.jp (P.P.P.); shigeon@jichi.ac.jp (S.N.); kmurata@jichi.ac.jp (K.M.); 2 Center for Experimental Medicine, Jichi Medical University School of Medicine, 3311-1 Yakushiji, Shimotsuke, Tochigi 329-0498, Japan; sakunita@jichi.ac.jp

**Keywords:** Hepatitis E virus, swine farm, seroprevalence, environmental samples, phylogenetic analysis, cell culture

## Abstract

Hepatitis E virus (HEV) causes acute or chronic hepatitis in humans. Pigs are the primary reservoir for zoonotic HEV genotypes 3 and 4 worldwide. This study investigated the infection dynamics and genomic mutations of HEV in domestic pigs on a farrow-to-finish pig farm in Japan between 2012 and 2021. A high prevalence of anti-HEV IgG antibodies was noted among pigs on this farm in 2012, when the survey started, and persisted for at least nine years. During 2012–2021, HEV RNA was detected in both serum and fecal samples, indicating active viral replication. Environmental samples, including slurry samples in manure pits, feces on the floor, floor and wall swabs in pens, and dust samples, also tested positive for HEV RNA, suggesting potential sources of infection within the farm environment. Indeed, pigs raised in HEV-contaminated houses had a higher rate of HEV infection than those in an HEV-free house. All 104 HEV strains belonged to subgenotype 3b, showing a gradual decrease in nucleotide identities over time. The 2012 (swEJM1201802S) and 2021 (swEJM2100729F) HEV strains shared 97.9% sequence identity over the entire genome. Importantly, the swEJM2100729F strain efficiently propagated in human hepatoma cells, demonstrating its infectivity. These findings contribute to our understanding of the prevalence, transmission dynamics, and genetic characteristics of HEV in domestic pigs, emphasizing the potential risks associated with HEV infections and are crucial for developing effective strategies to mitigate the risk of HEV infection in both animals and humans.

## 1. Introduction

Hepatitis E virus (HEV) is a prevalent pathogen responsible for acute or fulminant hepatitis E in humans. It poses a significant public health concern, as it can lead to chronic infection in individuals with compromised immune systems [1]. The HEV genome is composed of a single-stranded, positive-sense RNA molecule that closely resembles cellular mRNA, featuring a m7G cap and a poly-A tail. In the bloodstream, HEV exists as a quasi-enveloped virus, wrapped in a membrane cloaked, while in feces it is shed as a nonenveloped virus [2]. The HEV genome spans approximately 7.2 kilobases and contains short stretches of untranslated regions (UTRs) at both ends [3]. It also comprises three open reading frames (ORFs) known as ORF1–3. ORF1 encodes a nonstructural polyprotein responsible for viral replication, containing multiple functional domains [4,5]. ORF2 encodes the viral capsid protein, which can be produced in infectious, glycosylated, and cleaved forms. The glycosylated and cleaved forms are thought to serve as immunogenic decoys [6,7]. ORF3 encodes a small palmitoylated protein crucial for the secretion of infectious virions in exosomal membranes [2,8].

HEV belongs to the family *Hepeviridae,* which has rapidly expanded and is now divided into two subfamilies: *Orthohepevirinae* and the *Parahepevirinae*, the latter of which consists only of the *Piscihepevirus* genus, which infects fish [9], while the former encompasses the genera *Paslahepevirus* (mammalian viruses), *Avihepevirus* (bird viruses), *Rocahepevirus* (rodent viruses), and *Chirohepevirus* (bat viruses). The species *Paslahepevirus balayani* comprises eight genotypes of HEV, with genotypes 1–4 HEVs (HEV-1 to HEV-4) being the most important human pathogens. HEV-1 and HEV-2 exclusively infect humans through the fecal-oral route in developing countries with inadequate sanitation. These strains are highly pathogenic and can cause severe hepatitis, with pregnant women facing a fatality rate of up to 25% [10,11]. Conversely, HEV-3 and HEV-4 strains are zoonotic, primarily transmitted through the consumption of undercooked pork or game meat. They often manifest as asymptomatic infections but can lead to symptomatic acute hepatitis, acute-on-chronic liver failure, or extrahepatic manifestations [1,3]. Of note, infection of humans can occur by other viruses including camel HEV-7 [12] or the more distantly related rat HEV [13]. HEV-3, HEV-4, HEV-7, and rat HEV can persist in immunocompromised patients and cause chronic hepatitis, eventually leading to cirrhosis and liver failure [1,12,13,14]. HEV-3 is further classified into at least 14 subgenotypes (3a–3m, and 3ra), while HEV-4 is subdivided into at least 9 subgenotypes (4a–4i) [15].

The first strain of swine HEV was identified in pigs in the United States [16]. Since then, numerous strains of swine HEV (HEV-3 and HEV-4) have been reported in pigs across all swine-producing countries. Pigs serve as a major reservoir for HEV, constituting a significant source of zoonotic infections in humans [17]. The prevalence of anti-HEV IgG antibodies among domestic pigs worldwide ranges from approximately 20 to 100%. The presence of HEV RNA in domestic swine farms varies from 0 to 20% [18]. Notably, the prevalence rates differ among countries, regions, and even individual farms within a given country [19].

In Japan, nationwide cross-sectional studies have shown the presence of antibody-positive pigs in 21 prefectures and 109 out of 117 (93%) studied farms, indicating the widespread infection of HEV in pigs throughout the country. The overall prevalence of anti-HEV IgG antibodies in Japan is 57% (2242 out of 3925 pigs) [20]. However, the age of pigs is an important factor influencing the variability of HEV prevalence, with an increasing prevalence observed with age. Ongoing HEV infection is particularly prominent in pigs 2 to 5 months old, reaching its highest prevalence at 3 months old [20]. Based on a comprehensive nationwide survey on the prevalence of HEV infection among the general population in Japan, it was found that 5.3% (1167/22,027) tested positive for anti-HEV IgG antibodies [20]. Since coverage for the anti-HEV IgA detection assay kit was initiated by the government health insurance program in October 2011, the reported cases of hepatitis E in Japan have been consistently increasing. In recent years, the annual number of reported cases has ranged from 450 to 500. It is presumed that the majority of these cases are a result of consuming pig meat and edible offal [21], indicating the importance of studying infection dynamics at pig farms.

A comprehensive understanding of the risk factors associated with HEV infection at different stages of pig production can be obtained through prospective and longitudinal investigations. Therefore, the present study examined the infection dynamics of HEV in domestic pigs on a farrow-to-finish pig farm in Japan between 2012 and 2021 using a prospective and longitudinal approach. In addition, we explored potential sources of HEV infection within the farm environment and analyzed the genomic mutations of circulating HEV strains on the farm during the specified period.

## 2. Materials and Methods

### 2.1. Collection of Serum and Fecal Samples from Domestic Pigs

Serum and fecal samples were obtained from 268 domestic pigs on a farrow-to-finish swine farm located in the northern part of mainland Japan between 2012 and 2021 (Table 1). In 2012, serum samples were obtained from a total of 120 pigs, with 30 samples collected from pigs at 2, 3, 4, and 5 months old. In subsequent years, serum samples were obtained serially, involving 19 and 26 pigs in 2013, 69 pigs in 2014, and 11 pigs in 2019, ranging from 1 month to 6 months old. In addition, serum samples were obtained from 3 pigs at 2 and 3 months old in 2016. Individual rectal fecal samples were obtained from 20 pigs at 2 or 3 months old in 2021. Subsequently, 15% (*w*/*v*) suspensions were prepared using a previously described method [22]. In brief, fecal specimens (5 g each) obtained from pigs were suspended at a concentration of 15% in phosphate-buffered saline (PBS; pH 7.2; Thermo Fisher Scientific Inc., Waltham, MA, USA). The suspensions were then subjected to centrifugation at 1600× *g* for 30 min at 4 °C using a versatile refrigerated centrifuge (Eppendorf Himac Technologies Co., Ltd., Ibaraki, Japan), according to the previously described method [23], and the resulting supernatant was collected. The supernatant was subsequently spun down at 6200× *g* for 5 min at 4 °C using a high-speed refrigerated microcentrifuge (Tomy Seiko, Tokyo, Japan), yielding a clear supernatant. The serum samples and aliquots of fecal supernatants were stored at −80 °C until further analyses.

### 2.2. Environmental Samples

During the years 2016 and 2018, various environmental samples were collected for analysis. These samples included excreted feces found in the manure pit of each pig house and on the floors of pens, as well as swabs taken from the floors and walls. In addition, feed samples in and around the troughs in the pens and dust samples collected from filters of the mixing fans were obtained. Excreted fecal samples were collected into conical tubes and prepared as 15% suspensions as described above. Swab samples were obtained by manually wiping a 15 cm × 15 cm surface area of the floors (excluding areas with excreted feces) or walls (within a height of 30 cm from the floor) in 3 consecutive places using gauze wipes moistened with saline. The swabs were then immersed in 5 mL of saline. To extract the liquid, the swabs were manually squeezed, and the resulting liquid was briefly mixed with a vortex mixer and centrifuged at 6200× *g*. The resulting supernatant was used in subsequent RNA extraction. For the feed samples, 20 mL of saline (with a density of 0.7–1.3 g/mL) was added, and the mixture was suspended. The suspensions were then centrifuged at 6200× *g* for 5 min, and the resulting supernatant was used for HEV RNA detection. To collect dust samples from the filters (30 cm × 30 cm) of the mixing fans used for 2 days, each filter was placed in a vinyl bag containing 50 mL of saline, and gentle agitation was applied. The tubes containing the resulting liquid were briefly mixed with a vortex mixer and centrifuged at 6200× *g* for 5 min. The resulting supernatant was then used in the subsequent RNA extraction.

### 2.3. An Enzyme-Linked Immunosorbent Assay (ELISA) for Detecting Anti-HEV IgG Antibodies

To detect anti-HEV IgG in serum samples from pigs, an enzyme-linked immunosorbent assay (ELISA) was performed using purified recombinant ORF2 protein from the HE-J1 strain (genotype 4) that had been expressed in the silkworm pupae [24], as described previously [25]. The optical density (OD) value of 0.274 was used as the cutoff value for anti-HEV IgG in swine serum samples. Test samples with OD values equal to or greater than the cutoff value were considered to be positive for anti-HEV IgG. The specificity of the anti-HEV IgG assay was verified according to the level of absorption with the same recombinant HEV ORF2 protein (50 μg/mL at the final concentration) that was used as the antigen probe [25]. In brief, if the OD value of the tested sample was reduced by ≥70% after absorption with the recombinant ORF2 protein, the sample was considered positive for anti-HEV IgG.

### 2.4. Qualitative and Quantitative Detection of HEV RNA

All collected serum samples, fecal specimens from pigs, and environmental samples were subjected to screening for the presence of HEV RNA, employing both qualitative and quantitative methods. Total RNA was extracted from 100 μL of serum by employing TRIzol LS Reagent (Thermo Fisher Scientific Inc.) following the manufacturer’s instructions. Moreover, RNA molecules were extracted from 100 or 200 µL of 15% fecal suspensions, which included those sourced from the slurry in the manure pit and feces on the floor. In addition, supernatants of swab samples, anticipated to possess high concentrations of PCR inhibitors, were initially extracted utilizing the High Pure Viral RNA Kit (Roche Diagnostics K.K., Tokyo, Japan) as per the manufacturer’s instructions. Subsequently, the RNA in the extracted material was purified using TRIzol LS Reagent. Furthermore, supernatants (1.2 mL) from feed and dust samples were subjected to centrifugation (Optima TLX, Beckman Coulter, Inc., Brea, CA, USA) at 146,000× *g* for 1 h. The resultant pellets were then resuspended in distilled water and subsequently processed using the High Pure Viral RNA Kit and TRIzol LS Reagent following the aforementioned protocols.

The extracted RNA was reverse transcribed using SuperScript IV (Thermo Fisher Scientific Inc.) and then subjected to nested polymerase chain reaction (PCR) with primers derived from conserved regions within the ORF2 region, which are shared among the four major genotypes (1–4) of HEV, following the previously described method [24]. First-round PCR resulted in an amplification product of 506 base pairs (bp) (nucleotides [nt] 5912–6417 of M73218), and second-round PCR yielded a product of 457 bp (nt 5922–6378 of M73218). The products of second-round PCR were analyzed through agarose gel electrophoresis, and samples showing a visible band of 457 bp were considered positive for HEV RNA. The specificity of the reverse transcription (RT)-PCR assays was verified by a sequence analysis, as described below, and the sensitivity of RT-PCR was determined as previously described [26].

For the quantitation of HEV RNA, real-time RT-PCR was performed using a previously described method [27]. In brief, total RNA extracted from 100 μL of diluted serum samples, fecal suspensions, or environmental sample supernatants was subjected to real-time RT-PCR using the QuantiTect Probe RT-PCR Kit (Qiagen, Tokyo, Japan) on a LightCycler apparatus (Roche Diagnostics K.K.). The thermal cycler conditions included incubation at 50 °C for 20 min and initial denaturation at 95 °C for 15 min, followed by 45 cycles of denaturation at 95 °C for 1 s and annealing/extension at 60 °C for 60 s. The reproducibility of the quantitative assay was assessed by testing each sample in duplicate, and the mean value was used for analyses.

### 2.5. Amplification of the Full-Length HEV Genome

Total RNA was extracted from 3 mL of serum sample (swEJM1201802S), obtained from a pig with the highest HEV load (6.4 × 10^2^ copies/mL) in 2012 or from 200 μL of a 15% suspension of feces (swEJM2100729F) obtained from a pig with the highest HEV load (5.0 × 10^7^ copies/mL) in 2021. The extracted RNA was then subjected to cDNA synthesis, followed by nested PCR amplification of 10 or 3 overlapping regions, including the extreme 5′- and 3′-terminal regions. PCR amplification was performed using high-fidelity DNA polymerases (KOD FX Neo [Toyobo, Osaka, Japan], *TaKaRa LA Taq* with GC Buffer [TaKaRa Bio, Shiga, Japan]) and the primers indicated in Supplementary Tables S1 and S2, whose sequences were derived from areas that are well-conserved across all genotype 3 HEV strains, for which the entire genomic sequences are known, and those obtained during the amplification procedure, according to a previously described method [28]. The amplified regions excluding the primer sequences were nt 1–49 (49 nt), nt 37–505 (469 nt), nt 488–1526 (1039 nt), nt 1496–2460 (965 nt), nt 2407–3244 (838 nt), nt 3219–4343 (1125 nt), nt 4282–5328 (1047 nt), nt 5306–6083 (778 nt), nt 5969–6380 (412 nt), and nt 6392–7226 (835 nt) for the swEJM1201802S strain (Supplementary Figure S1) and nt 1–49 (49 nt), nt 37–3945 (3909 nt), and nt 3600–7226 (3627 nt) for the swEJM2100729F strain (Supplementary Figure S1).

The extreme 5′-end sequence (nt 1–49) was determined using a modified rapid amplification of cDNA ends (RACE) technique called RNA ligase-mediated RACE (RLM-RACE) with the First Choice RLM-RACE kit (Ambion, Austin, TX, USA), as described previously [29]. Amplification of the 3′-end sequence (nt 6392–7226 [835 nt] or nt 3600–7226 [3627 nt], excluding the poly[A] tail) was performed using the RACE method, as described previously [29].

### 2.6. The Determination and Analysis of Nucleotide Sequences

The amplification PCR products were purified using the FastGene Gel/PCR Extraction Kit (NIPPON Genetics, Ltd., Tokyo, Japan). The purified PCR products were then directly sequenced or cloned into the T-Vector pMD20 (TaKaRa Bio), followed by sequencing using an Applied Biosystems 3130xl Genetic Analyzer (Thermo Fisher Scientific Inc.) and the BigDye Terminator v3.1 Cycle Sequencing Kit (Thermo Fisher Scientific Inc.). The sequence analysis was conducted using the Genetyx software program (version 13.1.2; Genetyx Corp., Tokyo, Japan), and multiple alignments were generated using the MUSCLE software program, version 3.5 [30]. Neighbor-joining trees based on Jukes–Cantor distances were constructed using the 412-nt HEV ORF2 sequence (nt 5944–6355 of M73218) or the full-length HEV sequence, as implemented in the MEGA7 software program (version 7.0.26) [31]. The robustness of the clusters was assessed by performing 1000 bootstrap replicates, and branches with bootstrap values exceeding 70% were considered to be grouped together [31]. Reference sequences for 46 strains of genotypes 1–8 [15] were used for a comparison.

### 2.7. Inoculum Preparation for Cell Culture

The 15% fecal suspension obtained from a pig (swEJM2100729F), as described in Section 2.1, was diluted at ratios of 1:10, 1:100, or 1:1000 with PBS without magnesium and calcium (PBS[–]) containing 0.2% (*w*/*v*) bovine serum albumin (BSA; Sigma-Aldrich, St. Louis, MO, USA). Prior to inoculation with fecal suspension, the virus stocks underwent purification by passing them through microfilters with pore sizes of 0.45 and 0.22 μm (Millex-GV; Millipore Corp., Bedford, MA, USA).

### 2.8. Cell Culture and Virus Inoculation

Human hepatocarcinoma cells, PLC/PRF/5 (ATCC No. CRL-8024; American Type Culture Collection, Manassas, VA, USA) were cultured in Dulbecco’s modified Eagle medium (DMEM; GIBCO Cat. No.12800-058; Life Technologies Japan, Tokyo, Japan) supplemented with 10% (*v*/*v*) heat-inactivated fetal bovine serum (FBS; Thermo Fisher Scientific Inc.), 100 U/mL penicillin G, 100 μg/mL streptomycin, and 2.5 μg/mL amphotericin B (growth medium) at 37 °C in a humidified 5% CO_2_ atmosphere, as previously described [32,33]. For virus inoculation, cells (2.5 × 10^5^ cells/mL) in 2.0 mL of medium were added to each well (diameter of 3.5 cm) of a 6-well microplate (IWAKI, Tsukuba, Japan) 3 days before virus infection. The monolayers of cultured cells in the 6-well microplates were washed twice with 1 mL of PBS(–) and then 0.2 mL of the fecal suspension, prepared as described above (10^6^, 10^5^, or 10^4^ copies/well), was inoculated into each well. After 1 h of inoculation at room temperature in wells with PLC/PRF/5 cells, the solution was removed, and the wells were washed 5 times with 1 mL of PBS(–). Subsequently, 2 mL of growth medium was added to the wells with PLC/PRF/5 cells. The propagation was carried out at 35.5 °C in a humidified 5% CO_2_ atmosphere. Every other day, half (1 mL) of the culture medium was replaced with fresh medium, and the collected media were stored at −80 °C until virus titration. Triplicate sets of virus specimens were inoculated in parallel into the cultivated cells in a 6-well plate. The HEV load was determined for culture supernatants from the inoculated wells, as described above.

### 2.9. Western Blotting

Culture supernatants and cell lysates collected at 32 days postinoculation (dpi) were suspended in a 2× sodium dodecyl sulfate (SDS) buffer composed of 125 mM Tris-HCl (pH 6.8), 2.0% (*w*/*v*) SDS, 0.01% bromophenol blue, and 20% (*v*/*v*) glycerol. The samples were incubated at 97 °C for 5 min after the addition of 2.5% (*v*/*v*) 2-mercaptoethanol. The denatured samples were subjected to SDS-PAGE on a 7.5% or 12% polyacrylamide gel, followed by transfer onto a polyvinylidene difluoride (PVDF) membrane (Immobilon 0.45 μm; Merck Millipore, Darmstadt, Germany). The membrane was immersed in PBS with 0.1% (*v*/*v*) Tween-20 (PBS-T) containing 5% skim milk (BD Sciences, San Jose, CA, USA) and washed with PBS-T. Subsequently, the membrane was incubated at room temperature for 1 h with monoclonal antibodies (mAbs: 1 μg/mL) against HEV ORF2 (H6210) [27] or ORF3 (TA0536) [34] as the primary antibody (in PBS-T containing 2.5% skim milk). After washing, the membrane was incubated with ECL anti-mouse IgG, horseradish peroxidase-linked species-specific whole antibody from sheep (1:10,000; GE Healthcare Japan, Tokyo, Japan) at room temperature for 1 h and then examined using SuperSignal West Atto Ultimate Sensitivity Substrate (Thermo Fisher Scientific Inc.). Protein bands were visualized using the ImageQuant LAS 500 system (GE Healthcare Japan). Precision Plus Protein Dual Color Standards (Bio-Rad Laboratories, Hercules, CA, USA) were used as molecular weight markers.

### 2.10. Indirect Immunofluorescence

The swEJM2100729F-infected PLC/PRF/5 cells at 32nd dpi seeded into 8 well chamber slides (Watson, Tokyo, Japan) were subjected to immunofluorescence staining. The cells were fixed in 4% (*v*/*v*) paraformaldehyde at room temperature for 15 min and permeabilized in 0.2% (*v*/*v*) Triton X-100 at room temperature for 10 min. After washing with PBS(–), the fixed cells were blocked with 1% BAS in PBS(–) for 30 min. The cells were then incubated with an mAb (10 μg/mL in PBS[–] with 1% BSA) against HEV ORF2 (H6210) [27] or ORF3 (TA0536) [34] at 37 °C for 2 h. Following another round of washing with PBS(–), the cells were stained with Alexa Fluor 488-conjugated anti-mouse IgG (Molecular Probes, Thermo Fisher Scientific; 2 μg/mL in PBS[–] with 1% BSA) at 37 °C for 1 h. Nuclei were counterstained with 4,6-diamidino-2-phenylindole (DAPI; Roche Diagnostics K.K, Tokyo, Japan). Slide glasses were mounted with Fluoromount /Plus medium (Diagnostic BioSystems, Pleasanton, CA, USA) and then viewed under an FV1000 confocal laser microscope (Olympus, Tokyo, Japan).

### 2.11. Statistical Analyses

Statistical analyses were performed using the Statcel 3 software program (OMS Publishing Inc., Saitama, Japan). Spearman’s rank correlation coefficient test was used to evaluate the correlation between changes by year and age of peak antibody titer or age of viremia onset. In addition, the Mann–Whitney U-test was used to statistically evaluate the difference in the age of peak antibody titer or the age of viremia onset, depending on the presence of HEV antibodies in mother pigs. *p*-values of ≤0.05 were considered significant.

## 3. Results

### 3.1. Prevalence of Anti-HEV IgG Antibodies and HEV RNA among Domestic Pigs on a Swine Farm in 2012

**Figure 1:**
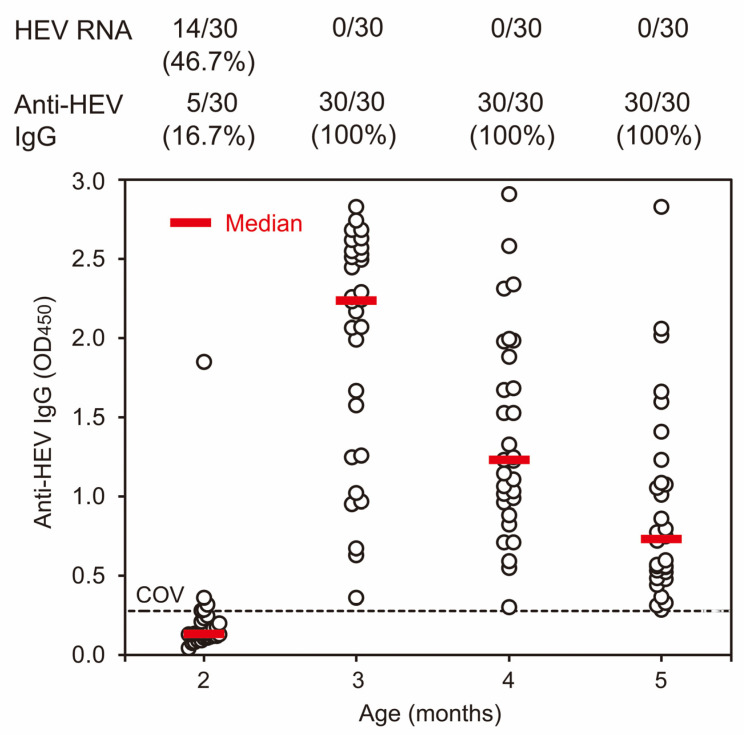
Distribution of the anti-HEV IgG OD value in serum samples of 2-, 3-, 4-, and 5-month-old pigs, obtained by the first cross-sectional study in 2012. Serum samples were obtained from 30 2-months-old pigs in the weaning house and 30 3-, 4-, and 5-months-old pigs in the growing house. The detection rates of HEV RNA and anti-HEV IgG are indicated at the top. The OD values are plotted at each age with open circles in the figure, accompanied by red horizontal bars indicating median values. The cutoff value (COV) of anti-HEV IgG is indicated by a dotted horizontal line.

At the initiation of the current investigation in 2012, serum samples were collected from 120 pigs on a swine farm to assess the presence of anti-HEV IgG antibodies. The results indicated that 95 pigs (79.2%) exhibited detectable HEV antibodies (Table 1), with OD values ranging from 0.277 to 2.909 (Figure 1). These antibodies were confirmed to be specific through an absorption test. Notably, the prevalence of HEV antibodies was 100% in pigs at 3, 4, and 5 months old. In addition, HEV RNA was only detectable in 46.7% (14/30) of 2-month-old pigs. Consequently, these findings establish a high prevalence of HEV infection among pigs on this investigated farm.

### 3.2. The Comparison of Kinetics of HEV Infection in Pigs in Weaning and Growing Houses with Varying HEV Contamination Statuses on a Swine Farm in 2013

During the 2013 study, pigs that were born and raised in the same farrowing house were subsequently transported to separate new and old houses. In the newly constructed house dedicated to weaning and growing (study group), only 2 out of 19 pigs (10.5%) tested positive for HEV RNA in their serum at 4 or 5 months of age. Conversely, in the weaning and growing houses known to be contaminated with HEV, where HEV-infected pigs had been previously reared (Control group), 10 out of 26 pigs (38.5%) were positive for HEV RNA in their serum by 3 months (Table 1).

**Figure 2:**
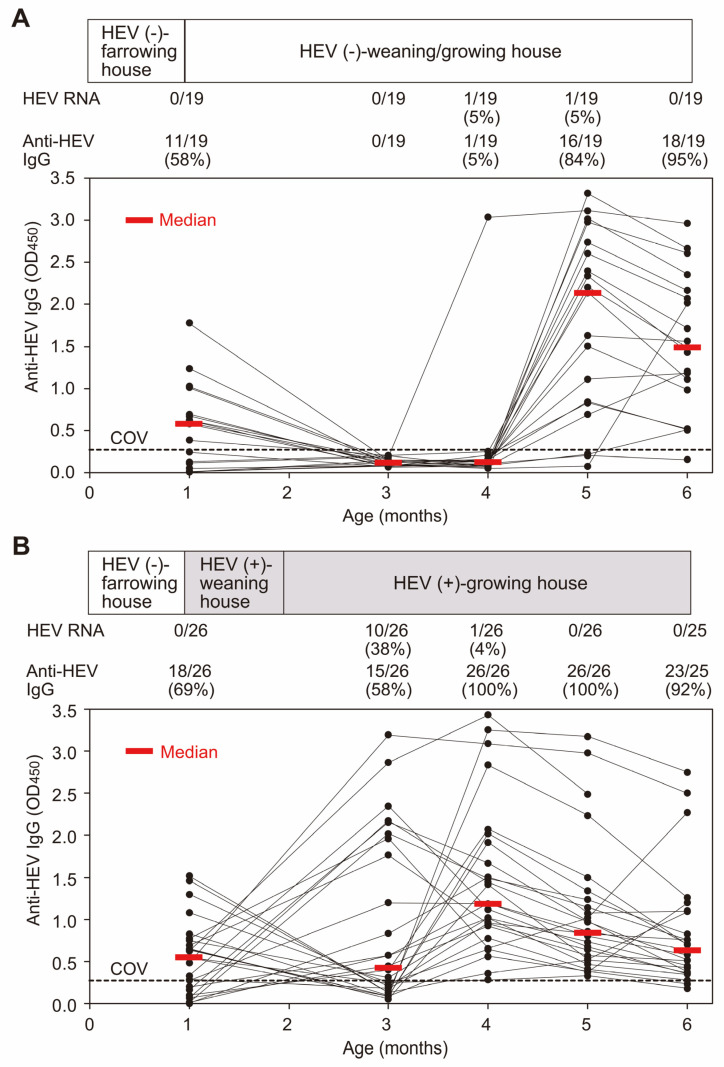
Changes in anti-HEV IgG OD values and the HEV RNA detectability rate in serial serum samples of 19 pigs in the study group (**A**) and 26 pigs (**B**) in the control group at 1–6 months old obtained by two cohort studies in 2013 (see Table 1). (**A**) A total of 19 pigs were raised in the farrowing house until they were 1 month old and then transferred to and raised in the newly constructed HEV-free house for weaning and growing from 1 to 6 months of age. (**B**) A total of 26 pigs were raised in the farrowing house until they were 1 month old and then transferred to and raised in the HEV-contaminated weaning house from 1 to 2 months, and then transferred to and raised in the HEV-contaminated growing house from 2 to 6 months old. The detection rates of HEV RNA and anti-HEV IgG are indicated below the open bar. The OD values are plotted at each age with closed circles in the figure at the bottom, accompanied by red horizontal bars indicating median values: closed circles connected by a line correspond to the data of one pig. The cutoff value (COV) of anti-HEV IgG is indicated by a dotted horizontal line.

Although nearly all pigs in both houses acquired HEV antibodies by the end of the six-month observation period, only 2 pigs (10.5%) in the study group exhibited detectable anti-HEV IgG at 4 months in the initial HEV-free house, with the median anti-HEV IgG level peaking at 5 months (Figure 2A). In contrast, all 26 pigs in the HEV-contaminated weaning and growing houses (Control group) tested positive for anti-HEV IgG by 4 months of age, and the median anti-HEV IgG level reached its highest point when they reached 4 months (Figure 2B).

### 3.3. Prospective Cohort Studies Conducted to Investigate the Highest HEV Antibody Response and the Initial Occurrence of HEV Viremia in Domestic Pigs on a Swine Farm in 2013, 2014, and 2019

Monthly serial serum samples were collected from a total of 26, 69, and 11 pigs over a period of 1 to 6 months (excluding 2 months in 2013) in 2013, 2014, and 2019, respectively. These samples were analyzed for the presence of anti-HEV IgG and HEV RNA, as shown in Table 2. The highest OD value of anti-HEV IgG was consistently observed at 4 months old across all 3 years. In addition, HEV RNA in serum was found to be positive in the majority of pigs (38–64%) at 3 months of age in all 3 years. Notably, in the 2014 study, there was no statistically significant difference observed between pigs born to sows with or without anti-HEV IgG regarding the months after birth showing the highest HEV antibody response or the first appearance of HEV RNA in serum. These findings suggest that the dynamics of HEV infection in pigs two to six months old remained relatively unchanged between 2013 and 2019 and were not influenced by the presence of maternal antibodies against HEV in pigs.

### 3.4. Consistent Detection of HEV RNA in Environmental Samples Collected from Weaning and Growing Houses on the Investigated Farm during 2016 and 2018

Fecal samples obtained from the manure-ditch in each house were tested for the presence of HEV RNA. While HEV RNA was not detected in fecal samples from farrowing, sow, or boar houses, it was consistently found in fecal samples from weaning and growing houses at a viral load of approximately 10^5^ copies/g (Table 3). Subsequently, fecal samples were collected from 3 to 10 independent pens within each house. Although none of the 10 fecal samples collected from the farrowing house tested positive for HEV RNA, fecal samples from both weaning and growing houses showed 100% (5/5) and 66.7% (2/3) positivity, respectively, with viral loads ranging from 1.8 × 10^2^ to 1.2 × 10^6^ copies/g (Table 3). Furthermore, floor and wall swabs obtained from the weaning and growing houses were tested for HEV RNA, and the virus was detected in both swab types, with the highest viral load recorded as 4.9 × 10^2^ copies/cm^2^ (Table 3). In addition, when feed samples from and around the trough in 4 pens of the weaning house were examined, HEV RNA was found at varying titers ranging from 1.8 × 10^1^ to 1.8 × 10^5^ copies/g. Interestingly, dust samples collected from filters of mixing fans in four rooms of the weaning house also contained detectable levels of HEV RNA, with the highest titer recorded as 8.0 × 10^5^ copies/filter, despite the filters being in use for only two days (Table 3).

### 3.5. The Comparison of Partial ORF2 Sequences among 104 HEV Strains Obtained from 2012 and 2021

**Figure 3:**
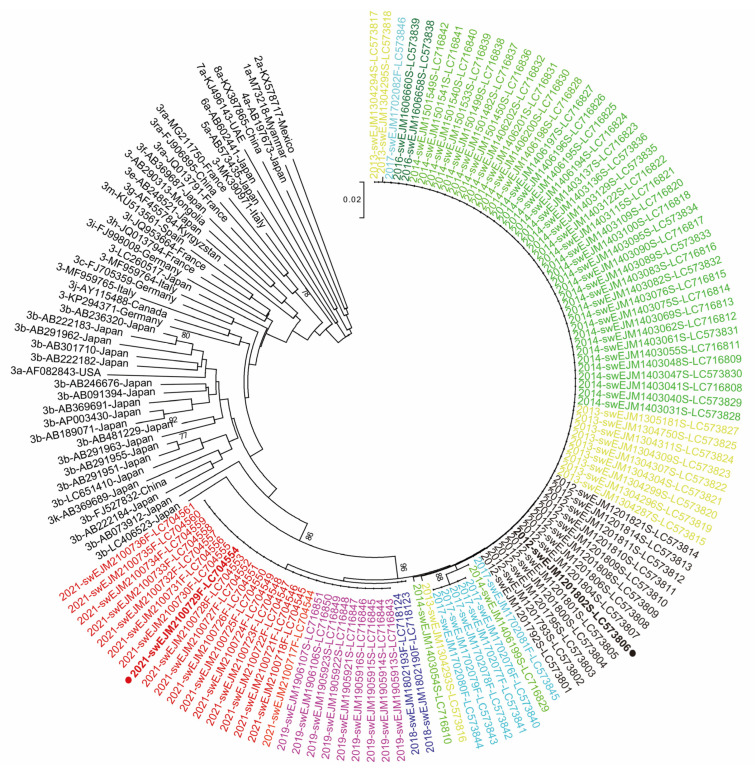
A phylogenetic tree of the partial ORF2 sequences of the HEV strains obtained from 104 pigs in the present study with 46 reported HEV sequences of genotypes 1–8, whose entire genomic sequences have been determined. A neighbor-joining tree of Jukes–Cantor distances was constructed based on the 412-nt ORF2 HEV sequences. The HEV strains obtained in the present study are shown with the year of isolation, followed by the isolate name and accession number and color coded by isolation year for visual clarity. The HEV strains whose entire genomic sequences were determined in the present study are marked with closed circles. Each reference sequence is shown with the genotype/subtype, followed by the accession number and name of the country in which it was detected. The bootstrap values (≥70%) of the nodes are indicated as a percentage of data obtained from 1000 resamplings. The scale bar represents the number of nucleotide substitutions per site.

In this study, we examined the partial ORF2 sequence (412 nt, excluding primer sequences at both ends) of 104 HEV strains obtained from serum or fecal samples collected between 2012 and 2021. The nucleotide sequence identity among these 104 HEV strains ranged from 96.3 to 100%. A phylogenetic analysis revealed that all strains formed a distinct cluster with a high bootstrap value of 96% (Figure 3).

To further investigate the evolutionary changes in HEV strains, we compared the nucleotide sequence identities between a representative HEV strain from 2012 (swEJM1201802S), whose complete genomic sequence was determined, and the remaining 103 HEV strains obtained during the 9-year observation period (Table 4). The results showed a gradual decrease in sequence identities over time. In 2012, the mean identity was 99.6%, which decreased to 96.9–97.0% in 2019 and 2021. Similarly, the median identity decreased from 99.7% in 2012 to 97.0% in 2019 and 2021. Furthermore, we observed the emergence of mutations in the HEV strains over time, with mutations detected at 11 nucleotide positions that had been conserved in the 2012 strains. The number of nucleotides with substitutions of more than half of the original base gradually increased from 1 nt in 2014 to 2 nt in 2016 and 2017, 7 nt in 2018, 10 nt in 2019, and 11 nt in 2021. These findings indicate the accumulation of nucleotide mutations during the observation period. However, the 11 nucleotide substitutions identified were not associated with any amino acid mutations, most likely due to the high level of conservation observed in the ORF2 (capsid) protein.

### 3.6. The Comparison of Entire Genomic Sequences of Representative HEV Strains from 2012 and 2021

To compare the entire genomic sequences of two representative HEV strains from 2012 and 2021, we determined the full-length nucleotide sequences of the swEJM1201802S strain from 2012 and the swEJM2100729F strain from 2021. Both strains had a genomic length of 7226 nt (excluding the poly[A] tract at the 3′ terminus) and contained three major ORFs, consistent with HEV isolates reported in humans and other animals [35]. The 5′UTR sequences of the swEJM1201802S and swEJM2100729F strains consisted of 25 nt, while their 3′UTR sequences comprised 75 nt (excluding the poly[A] tail).

**Figure 4:**
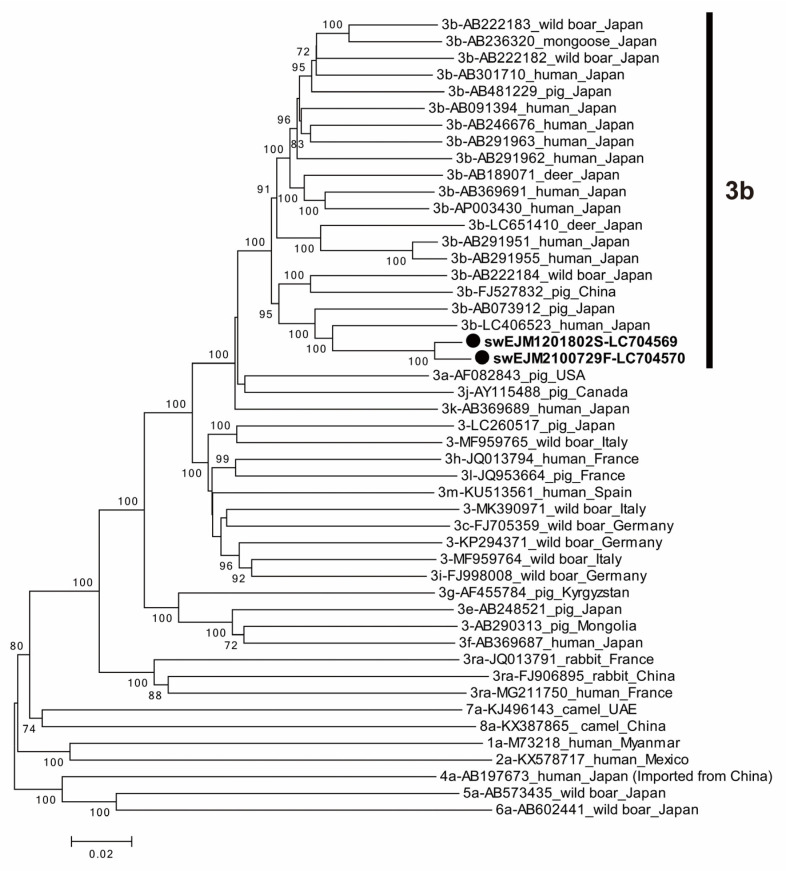
A phylogenetic tree of the entire genomic sequence of the HEV strains (swEJM1201802S and swEJM2100729F) obtained in the present study with 46 reference sequences of genotypes 1–8, whose entire genomic sequences have been determined. Each reference sequence is shown with genotype/subtype, followed by the accession number, the species of the animal from which it was detected, and the name of the country in which it was detected. The HEV strains obtained in the present study are highlighted with closed circles and indicated in bold, followed by the accession number. The HEV strains, which were classifiable into subtype 3b, are indicated with a vertical bar. The bootstrap values (≥70%) of the nodes are indicated as a percentage of data obtained from 1000 resamplings. The scale bar represents the number of nucleotide substitutions per site.

A comparative analysis revealed a 97.9% sequence identity between the swEJM1201802S and swEJM2100729F strains across the entire genome. Within specific ORFs, the nucleotide sequence identities were 98.0%, 97.5%, and 99.1% for ORF1, ORF2, and ORF3, respectively. In addition, the amino acid sequences exhibited a shared identity of 99.6% in ORF1, 99.7% in ORF2, and 99.1% in ORF3 (Table 5). A phylogenetic analysis based on the entire genome (Figure 4) demonstrated that the swEJM1201802S and swEJM2100729F strains formed a cluster with the reported 3b strains, supported by a bootstrap value of 100%, confirming segregation into subgenotype 3b. It is worth noting that subgenotype 3b is indigenous to Japan, as represented by Figure 4. However, the overall similarity with the reported 3b strains was relatively low, ranging from 87.6% to 91.7%. The most closely related HEV-3b strain was recovered from a Japanese patient with hepatitis E (LC406523) (Figure 4).

### 3.7. The swEJM2100729F Strain Efficiently Propagated in Human Hepatoma Cells

**Figure 5:**
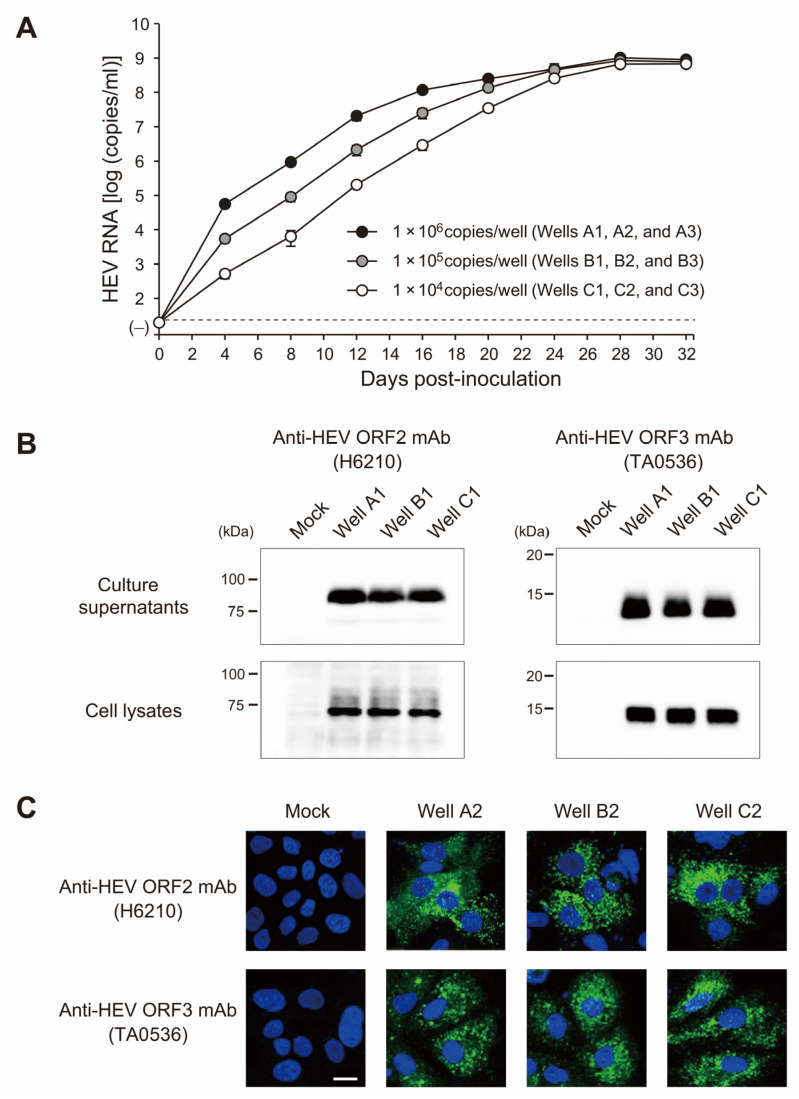
Characteristics of the swEJM2100729F strain obtained in the present study. (**A**) Quantitation of HEV RNA in the culture supernatant of PLC/PRF/5 cells inoculated with fecal supernatants of the swEJM2100729F strain with the indicated viral loads. The HEV RNA titer is plotted as the mean ± SD of three wells. (**B**) Western blotting of the HEV ORF2 (upper panel) and ORF3 (lower panel) proteins of the swEJM2100729F strain in culture supernatants and cell lysates with the indicated mAbs (see (**A**) for wells A–C at 32 dpi). (**C**) Indirect immunofluorescence staining of the HEV ORF2 (upper panel) and ORF3 (lower panel) proteins in PLC/PRF/5 cells. PLC/PRF/5 cells inoculated with culture supernatants of the swEJM2100729F strain (see (**A**) for wells A–C at 32 dpi) were incubated with the indicated mAbs and then stained with Alexa Fluor-488-conjugated anti-mouse IgG. Mock-infected cells were used as a negative control. Nuclei were stained with DAPI. Merged signals are shown. Bar 20 μm.

A fecal suspension containing the swEJM2100729F strain with the highest titer of HEV RNA, whose complete genomic sequence was determined as described above, was utilized in the experiment. The suspension was inoculated onto PLC/PRF/5 cells in a 6-well microplate at HEV RNA titers of 1.0 × 10^4^, 1.0 × 10^5^, and 1.0 × 10^6^ copies/well (Figure 5A). In a dose-dependent manner, the HEV RNA titer progressively increased, resulting in an HEV load of >10^9^ copies/mL at 32 dpi. The efficient propagation of the swine HEV strain was confirmed by Western blotting, which detected the presence of ORF2 and ORF3 proteins (Figure 5B), as well as by an immunofluorescence analysis (Figure 5C). The observed variation in the length of ORF2 proteins in the culture supernatants and cell lysates can be attributed to the presence and extent of glycosylation of the viral protein [36]. The glycosylated secreted form of the ORF2 protein is predominantly detectable in the culture medium [6,7].

## 4. Discussion

We obtained a number of important findings in the present study. First, the persistence of infection from the same HEV strain of subgenotype 3b lasted for at least nine years on a farrow-to-finish pig farm. This farm exhibited a high prevalence of HEV infection among domestic pigs in 2012, marking the beginning of the survey. Second, the contamination status of HEV within the farmhouses influenced the infection dynamics, as pigs reared in HEV-contaminated houses displayed a higher rate of HEV infection and manifested viremia at an earlier stage than those residing in a newly constructed HEV-free house. Third, environmental samples collected from weaning and growing houses consistently contained HEV RNA, suggesting potential sources of viral transmission. Fourth, a sequence analysis of HEV strains unveiled a gradual decrease in sequence identities over time, concomitant with the emergence of nucleotide mutations. Fifth and most importantly, the 2021 HEV strain (swEJM2100729F), obtained from feces of an infected pig in 2021, efficiently propagated in human hepatoma cells, thus demonstrating its infectivity. These findings will help improve our understanding of the prevalence, transmission dynamics, and genetic characteristics of HEV in domestic pigs, underscoring the potential risks associated with HEV infection in both animals and humans. The sequencing of entire HEV genomes obtained from pigs with a time gap of nine years on the identical farm, coupled with the successful cultivation of one of the swine HEV strains derived from a fecal sample, adds significant originality to our present research.

The findings in the 2012 study indicated a high prevalence of HEV infection among pigs on the investigated farm, with the presence of anti-HEV IgG antibodies being noted in 100% of 6-month-old pigs and that of HEV RNA in 46.7% of 2-month-old pigs (Figure 1, Table 1), similar to pigs on nearly all farms in Japan [25,37,38,39]. These results establish a significant burden of HEV infection in the pig population on this investigated farm. Although this study was a survey conducted on a single farm, it will help us understand the infection status of most pig farms, where HEV is persistently present, in Japan.

In 2012, when the present survey started, HEV RNA was detected exclusively in 2-month-old pigs, while in 2013 and later, it was detectable most frequently in 3-month-old pigs (Table 2). While the exact reason for this shift is unclear, it is speculated to be because the disinfectant used in the pig houses was changed in 2013 from a bactericidal disinfectant containing didecyldimethylammonium chloride (Cleanyell; Kyoritsu Seiyaku, Tokyo, Japan) to a combined hypochlorous acid disinfectant containing potassium hydrogen peroxymonosulfate (Antec Virkon S; Elanco Japan, Tokyo, Japan). Although it has been reported that the combined hypochlorous acid disinfectant can inactivate African swine fever virus at various concentrations in vitro [40], the disinfectant did not show sufficient virucidal efficacy for HEV in the environment. Indeed, in prospective cohort studies conducted over several years (2013, 2014, and 2019), the highest OD value of anti-HEV IgG was consistently observed at 4 months. In addition, in 2019, 2 of 11 pigs had HEV RNA first detected at 4 months, but HEV RNA was detected in the majority of pigs (38–64%) at 3 months in 2013, 2014, and 2019 (Table 2). In support of our observation, HEV RNA was detectable from floor and wall swabs even after washing of the floors and walls with water and spraying pens with disinfectants.

A few longitudinal studies have been conducted to assess the dynamics of infection in pigs [37,39,41,42,43,44,45]. The differences observed in these studies confirmed that several factors may influence the occurrence of the infection in pigs, its persistence during a pig’s life, and the dynamics of infection [41]. The present longitudinal studies conducted in 2013 confirmed that the contamination status of HEV in weaning and growing houses affected the dynamics of infection. Pigs in the HEV-contaminated houses had a higher rate of infection than those in the HEV-free house, with 38.5% (10/26) testing positive for HEV RNA by 3 months versus only 10.5% (2/19) in the HEV-free house. Since neither the farrowing house nor the newly constructed house for weaning and growing were contaminated with HEV, the first HEV-infected pig, which showed high-titer anti-HEV IgG at four months, was presumed to have accidentally contracted HEV infection while being transported via vehicle from the farrowing house to the HEV-free house. In addition, since seroconversion and HEV viremia occurred three months after the transfer, the possibility of the infection being brought in via imported items or workers or invading from the surrounding environment cannot be denied either.

Pigs possess an epithelial-chorial placenta that can prevent IgG from sows being transferred to piglets, but newborn piglets are able to acquire passive immunity through colostrum [44]. Passive immunization is the primary protection against infection in early life for several animal species, including pigs, and the importance of maternally derived antibodies for protection against early life HEV infection in pigs has been reported [46]. Notably, the presence of HEV antibodies in sows did not significantly affect the timing of the highest antibody response or the first appearance of HEV RNA in serum in the piglets in the present study (Table 2). Protection by maternally derived antibodies is known to be only partial and temporary due to antibody waning [47]. It is presumed that exposure to HEV infection in pigs on our studied farm occurred at a time when antibody titers declined and infection could not be sufficiently prevented. Supporting this speculation, only 1 (1.4%) of 69 two-month-old pigs was positive for anti-HEV IgG (Table 1).

Domestic pigs constitute the main reservoir of zoonotic HEV genotypes 3 and 4. However, more information about its origins and pathways in pig herds is needed to establish measures to prevent further transmissions. As HEV excretion predominantly occurs via feces, it has been reported that manure storage, fecal-contaminated housings and fomites, feed, and drinking water are potential sources of infection [41]. The present study also demonstrated the presence of HEV in different environmental samples, including slurry samples in manure pits, feces on the floor, floor swabs, wall swabs, feed samples, and dust samples (Table 3). HEV RNA was consistently detected in fecal samples from weaning and growing houses, as well as in floor and wall swabs from these houses. HEV RNA was even found in feed samples and dust samples collected from the weaning house. These results indicate that the contamination status of HEV in the houses may influence the dynamics of HEV infection in pigs during the weaning and growing stages, even within the same farm, and suggest that the virus is widely present in the pig’s living environment, indicating the potential for environmental contamination, leading to continual herd reinfection and thus serving as a persistent source of infection, as described previously [48,49,50]. The presence of HEV in the environment highlights the need for comprehensive hygiene and biosecurity measures to minimize the risk of transmission.

The comparison of partial and entire genomic sequences of HEV strains collected over several years reveals evolutionary changes in the virus. Sequence analyses demonstrated a gradual decrease in nucleotide sequence identities over time, indicating the accumulation of mutations in the HEV strains. The emergence of mutations in previously conserved nucleotide positions, as indicated for partial ORF2 sequences in Table 4, suggests the ongoing evolution of the virus. High variability and frequent selection of mutations in the HEV genome is due to the transcription process [51]. The HEV mutation rates have been estimated indirectly from clinical isolates as 1.40–1.72 × 10^−3^ base substitutions per site per year [52]. Comparable mutation rates have been observed during propagation and consecutive passages of HEV strains for adaptation to cell culture (0.87–2.71 × 10^−3^ base substitutions per site per year) [53,54]. Based on a set of 2 HEV strains (swEJM1201802S and swEJM2100729F) isolated 9 years apart from infected pigs on the same farm, exhibiting a 2.1% difference over the entire genome, the mutation rate was estimated to be 2.38 × 10^−3^ base substitutions per site per year, with the same order as those reported for hepatitis C viruses in a persistently infected chimpanzee (1.44 × 10^−3^ base substitutions per site per year) [55]. These findings highlight the genetic variability of HEV and provide insights into its evolutionary dynamics over a nine-year observation period. Previous studies have demonstrated disparities in viral genome sequences between HEV isolates obtained from both fecal and serum samples of identical patients [56]. As a limitation of this study, it is important to acknowledge that the finding regarding genomic mutations and evolutionary changes require further verification. This is due to the analysis and comparison of only two individual viral genomes, one from a serum sample in 2012 and the other from a fecal sample in 2021.

The current study also investigated the ability of a swine HEV strain (swEJM2100729F) obtained from feces of a pig in 2021 to propagate in human hepatoma cells. The results demonstrated the efficient propagation of the swine HEV strain, as evidenced by the progressive increase in HEV RNA titer over time, consistent with previous studies [48,57]. The presence of viral proteins ORF2 and ORF3 confirmed the replication of the virus in human cells. This finding raises concerns about the potential zoonotic transmission of swine HEV to humans and underscores the importance of monitoring and preventing such transmissions. It would be intriguing to investigate whether viral isolates, collected from fecal samples obtained prior to 2021, have exhibited a net gain or loss in overall fitness over the course of several years. Furthermore, exploring the potential impact of identified amino acid changes on viral fitness would be of great significance.

In order to minimize the risk of human infection, it is crucial to ensure that pigs test negative for HEV at the time they are sent to the slaughterhouse. It is of utmost importance to thoroughly examine the absence of HEV in pigs, particularly in the liver, before they are transported from the farm. According to a study conducted in 2019, rectal fecal samples from all 11 pigs at 6 months tested negative for HEV RNA. This suggests that the pigs designated for slaughter at the investigated farm were devoid of HEV, thus contributing to the reduction in HEV transmission to humans.

In conclusion, this study on HEV infection in pigs provides valuable scientific insights into the prevalence, dynamics, and evolutionary changes of the virus. The findings contribute to our understanding of the interplay between HEV and pigs, highlighting the need for robust surveillance, improved biosecurity measures, and public health interventions to minimize the risk of zoonotic transmission. By addressing this important public health issue, our study’s findings have implications for both the livestock industry and human health.

## Figures and Tables

**Table 1:** Prevalence of HEV infection in domestic pigs on an HEV-prevalent pig farm during 2012–2021.

Year	Study Type	Sample	Age in Months ^a^ (Days after Birth)	Place (House)	Number. of Pigs	Number (%) of Pigs with:	
						Anti-HEV IgG	HEV RNA
2012	Cross-sectional	Serum	2 (60–63)	Weaning	30	5 (16.7)	14 (46.7)
			3 (90–93)	Growing	30	30 (100)	0
			4 (121–126)	Growing	30	30 (100)	0
			5 (149–155)	Growing	30	30 (100)	0
			Subtotal		120	95 (79.2)	14 (11.7)
2013	Cohort (Study group)	Serum	1 (19–23)	Farrowing	19	11 (57.9)	0
			3 (82–86)	HEV-free (A) ^b^	19	0	0
			4 (110–114)	HEV-free (A)	19	1 (5.3)	1 (5.3)
			5 (138–142)	HEV-free (A)	19	16 (84.2)	1 (5.3)
			6 (166–170)	HEV-free (A)	19	18 (94.7)	0
			Subtotal		19	18 (94.7)	2 (10.5)
	Cohort (Control group)	Serum	1 (19–23)	Farrowing	26	18 (69.2)	0
			3 (82–86)	Growing	26	15 (57.7)	10 (38.5)
			4 (110–114)	Growing	26	26 (100)	1 (3.8)
			5 (138–142)	Growing	26	26 (100)	0
			6 (166–170)	Growing	25	23 (92.0)	0
			Subtotal		26	26 (100)	10 (38.5)
2014	Cohort	Serum	1 (22–27)	Farrowing	69	23 (33.3)	0
			2 (53–60)	Weaning	69	1 (1.4)	2 (2.9)
			3 (83–91)	Growing	69	54 (78.3)	39 (56.5)
			4 (113–123)	Growing	69	69 (100)	3 (4.3)
			5 (144–156)	Growing	69	68 (98.6)	0
			6 (173–207)	Growing	69	67 (97.1)	0
			Subtotal		69	69 (100)	40 (58.0)
2016	Cohort	Serum	2 (58–59)	HEV-free (B) ^c^	3	1 (33.3)	2 (66.7)
			3 (85–86)	HEV-free (B)	3	3 (100)	2 (66.7)
			Subtotal		3	3 (100)	2 (66.7)
2019	Cohort	Serum	1 (24–28)	Farrowing	11	6 (54.5)	0
			2 (54–58)	Growing ^d^	11	2 (18.2)	0
			3 (88–92)	Growing	11	2 (18.2)	7 (63.6)
			4 (117–121)	Growing	11	7 (63.6)	2 (18.2)
			5 (144–148)	Growing	11	10 (90.9)	0
			6 (173–177)	Growing	11	10 (90.9)	0
			Subtotal		11	11 (100)	9 (81.8)
2021	Cross-sectional	Feces	2 ^e^	Weaning	10	-	8 (80.0)
			3 ^e^	Growing	10	-	10 (100)
			Subtotal		20	-	18 (90.0)
			Total		268	222 (89.5) ^f^	95 (35.4)

HEV, hepatitis E virus. ^a^ “1 month old” stands for 19–28 days after birth, “2 months old” for 53–63 days, “3 months old” for 82–93 days, “4 months old” for 110–126 days, “5 months old” for 138–156 days, and “6 months old” for 166–207 days. ^b^ A total of 19 pigs in a farrowing house were transferred to and raised in a newly built HEV-free weaning/growing house on the same farm. ^c^ Three pigs in a weaning house were transferred to and raised in another HEV-free weaning/growing house located outside of the farm. ^d^ These pigs were raised in a growing house even though they were 54–58 days old. ^e^ The time since birth was not available. ^f^ Serum samples were collected from a total of 248 pigs.

**Table 2:** Three cohort studies on the highest HEV antibody response and the first appearance of HEV viremia in domestic pigs on a swine farm, conducted in 2013, 2014 and 2019.

Year	Number of Pigs Studied		Months after Birth						*p*-Value
			1	2	3	4	5	6	
Number (%) of pigs by month with the highest OD value of anti-HEV IgG									
2013 ^a^	26		18 (69.2)	NA ^b^	7 (26.9)	15 (57.7)	2 (7.7)	2 (7.7)	(a) 0.9760 (c) 0.1583
2014 ^a^	69		23 (33.3)	0	31 (44.9)	31 (44.9)	5 (7.2)	2 (2.9)	
	With ^c^	50	23 (46.0)	0	20 (40.0)	24 (48.0)	4 (8.0)	2 (4.0)	
	Without ^c^	19	0	0	11 (57.9)	7 (36.8)	1 (5.3)	0	
2019 ^a^	11		6 (54.5)	0	2 (18.2)	5 (45.5)	3 (27.3)	1 (9.1)	
Number (%) of pigs by month with the first appearance of HEV viremia									
2013 ^d^	26		0	NA	10 (38.5)	0	0	0	(d) 0.0837 (e) 0.4081
2014 ^d^	69		0	2 (2.9)	38 (55.1)	0	0	0	
	With ^e^	50	0	1 (2.0)	29 (58.0)	0	0	0	
	Without ^e^	19	0	1 (5.3)	9 (47.4)	0	0	0	
2019 ^d^	11		0	0	7 (63.6)	2 (18.2)	0	0	

HEV, hepatitis E virus; OD, optical density. ^a^ Comparison among pigs in 2013, 2014 and 2019 (Spearman’s rank correlation test). ^b^ NA, not available. ^c^ Between pigs whose sow had (with) or did not have (without) anti-HEV IgG (Mann–Whitney U-test). ^d^ Comparison among pigs in 2013, 2014 and 2019 (Spearman’s rank correlation test). ^e^ Between pigs whose sow had (with) or did not have (without) anti-HEV IgG (Mann–Whitney U-test).

**Table 3:** Detection of HEV RNA in different environmental samples obtained in different houses on a swine farm during 2016 and 2018.

Place	Slurry in the Manure-Pit of Each House (Copies/g) ^a^	Samples in Pens				Dust Samples from Filters in Four Rooms (Copies/Filter) ^e^
		Feces on the Floor ^b^	Floor Swab (Copies/cm^2^) ^c^	Wall Swab (Copies/cm^2^) ^c^	Feed Samples from/around the Trough in One Each Pen of Four Rooms (Copies/g) ^d^	
Farrowing house	(–) ^f^	0/10	(–)	(–)	NT ^g^	NT
Weaning house	2.7 × 10^5^	5/5 (100%) ^h^	4.9 × 10^2^	2.2 × 10^2^	(R1) 4.5 × 10^1^/7.3 × 10^2^ (R2) 1.8 × 10^1^/7.7 × 10^2^ (R3) 3.7 × 10^3^/1.8 × 10^5^ (R4) 1.6 × 10^3^/9.9 × 10^4^	(R1) 3.0 × 10^4^ (R2) 3.4 × 10^3^ (R3) 7.5 × 10^5^ (R4) 8.0 × 10^5^
Growing house	7.4 × 10^5^	2/3 (67%) ^i^	(+) ^j^	(+) ^j^	NT	NT
Sow house	(–)	NT	NT	NT	NT	NT
Boar house	(–)	NT	NT	NT	NT	NT

HEV, hepatitis E virus. ^a^ HEV in the slurry samples collected in 2018 was subjected to nucleotide sequencing (see Figure 3). ^b^ HEV in the feces collected in 2017 was subjected to nucleotide sequencing (see Figure 3). ^c^ A surface size of 15 cm × 15 cm of unclean floors or walls was wiped manually in three consecutive places with a gauze moistened with saline in 2016. The swabs were immersed in 5 mL of saline and squeezed by sterilized stick to obtain the liquid. The resulting liquid was subjected to the detection of HEV RNA. ^d^ Feed samples obtained in each pen of four rooms (R1 to R4) in 2017 were combined with 20 mL of saline (0.7–1.3 g/mL) and suspended. The suspensions were then centrifuged, and the resulting supernatants were used for the detection of HEV RNA. ^e^ Each filter (30 cm × 30 cm) of mixing fans in four rooms (R1 to R4) was removed and immersed in 50 mL of saline in a vinyl bag. The filters were squeezed by hand to obtain the liquid. The resulting liquid was subjected to the detection of HEV RNA. ^f^ Negative for HEV RNA. ^g^ Not tested. ^h^ With HEV RNA titer (mean: 4.4 × 10^5^; range: 1.1 × 10^4^–1.2 × 10^6^ copies/g). ^i^ With HEV RNA titer (mean: 1.2 × 10^3^; range: 1.8 × 10^2^–2.2 × 10^3^ copies/g). ^j^ HEV RNA was detectable by qualitative RT-PCR but not by quantitative reverse transcription-polymerase chain reaction.

**Table 4:** A comparison of partial ORF2 sequences between a representative HEV strain in 2012 (swEJM1201802S) whose entire genomic sequence was determined and HEV strains obtained during 2012 and 2021.

Year	Number of Strains Compared	Nucleotide Sequence Identity (%)		Number (%) of Strains with the Mutation at the Indicated Nucleotide Position ^a^:										
		Mean ± SD (Median)	Range	5972 T/C	6005 T/C	6035 T/C	6102 T/C	6116 T/C	6194 T/A	6266 T/C	6285 T/C	6287 G/A	6318 T/C	6344 T/C
2012	13	99.6 ± 0.1 (99.7)	99.5–99.7	0	0	0	0	0	0	0	0	0	0	0
2013	12	99.0 ± 0.5 (99.0)	98.0–99.5	0	0	0	0	0	0	0	0	0	0	0
2014	40	98.6 ± 0.5 (98.7)	97.8–99.2	0	0	5 (12.5)	19 (47.5)	1(2.5)	0	19 (47.5)	25 (62.5)	0	0	0
2016	2	98.0 ± 1.0 (98.0)	97.3–98.7	0	0	0	2 (100)	0	0	0	2 (100)	0	0	0
2017	7	98.2 ± 0.4 (98.0)	98.0–99.2	0	0	0	7 (100)	6 (85.7)	0	0	2 (28.6)	0	3 (42.9)	0
2018	2	97.5 ± 0.0 (97.5)	97.5	0	0	0	2 (100)	2 (100)	0	2 (100)	2 (100)	2 (100)	2 (100)	2 (100)
2019	9	96.9 ± 0.4 (97.0)	96.3–97.3	9 (100)	9 (100)	9 (100)	9 (100)	9 (100)	0	9 (100)	9 (100)	9 (100)	9 (100)	9 (100)
2021	18	97.0 ± 0.2 (97.0)	96.6–97.3	18 (100)	18 (100)	18 (100)	18 (100)	18 (100)	18 (100)	18 (100)	18 (100)	18 (100)	11 (61.1)	18 (100)

HEV, hepatitis E virus; SD, standard deviation. ^a^ Eleven mutated nucleotides in the 2021 strains that are not found in the 2012 strains.

**Table 5:** The comparison of the identity (%) over the entire genome, and the 5′UTR, ORF1, ORF2, ORF3, or 3′UTR sequences of the swEJM1201802S and swEJM2100729F strains obtained in the present study.

Region	Identity (%)	
	Nucleotides	Amino Acids
Entire genome ^a^	97.9 (7071/7226)	- ^b^
5′UTR	100 (25/25)	-
ORF1	98.0 (5006/5109)	99.6 (1696/1703) ^c^
ORF2	97.5 (1930/1980)	99.7 (658/660) ^d^
ORF3	99.1 (336/339)	99.1 (112/113) ^e^
3′UTR ^a^	97.3 (73/75)	-

UTR, untranslated region. ^a^ The poly(A) tail is excluded. ^b^ Not applicable. ^c^ Seven mutations are: S623C, N624G, T721A, H722Y, A870V, I1688V, and E1702V. ^d^ Two mutations are: A64T and E470V. ^e^ One mutation is: L78R.

## Data Availability

The nucleotide sequence data reported in this study have been assigned DDBJ/EMBL/GenBank accession numbers LC704569–LC704570 (full genome sequences) and LC573801–LC573848, LC704544–LC704561, LC716808–LC716851, and LC718123–LC718124 (partial ORF2 sequences).

## Supplementary Materials

The following supporting information can be downloaded at: https://www.mdpi.com/article/10.3390/v15071516/s1, Figure S1: The genomic organization of HEV and the regions amplified for nucleotide sequencing of the swEJM1201802S and swEJM2100729F genomes; Table S1: Primers and the adaptor used for full-genome sequencing of swEJM1201802S; Table S2: Primers and the adaptor used for full-genome sequencing of swEJM2100729F.
