# Supplementary material for: Infection Dynamics and Genomic Mutations of Hepatitis E Virus in Naturally Infected Pigs on a Farrow-to-Finish Farm in Japan: A Survey from 2012 to 2021
Source: Viruses. 2023 Jul 7;15(7):1516. doi: 10.3390/v15071516 (PMC10385168; doi:10.3390/v15071516)
Supplement: Supplementary file 1 [file viruses-15-01516-s001.zip › viruses-2493915-supplementary.pdf]

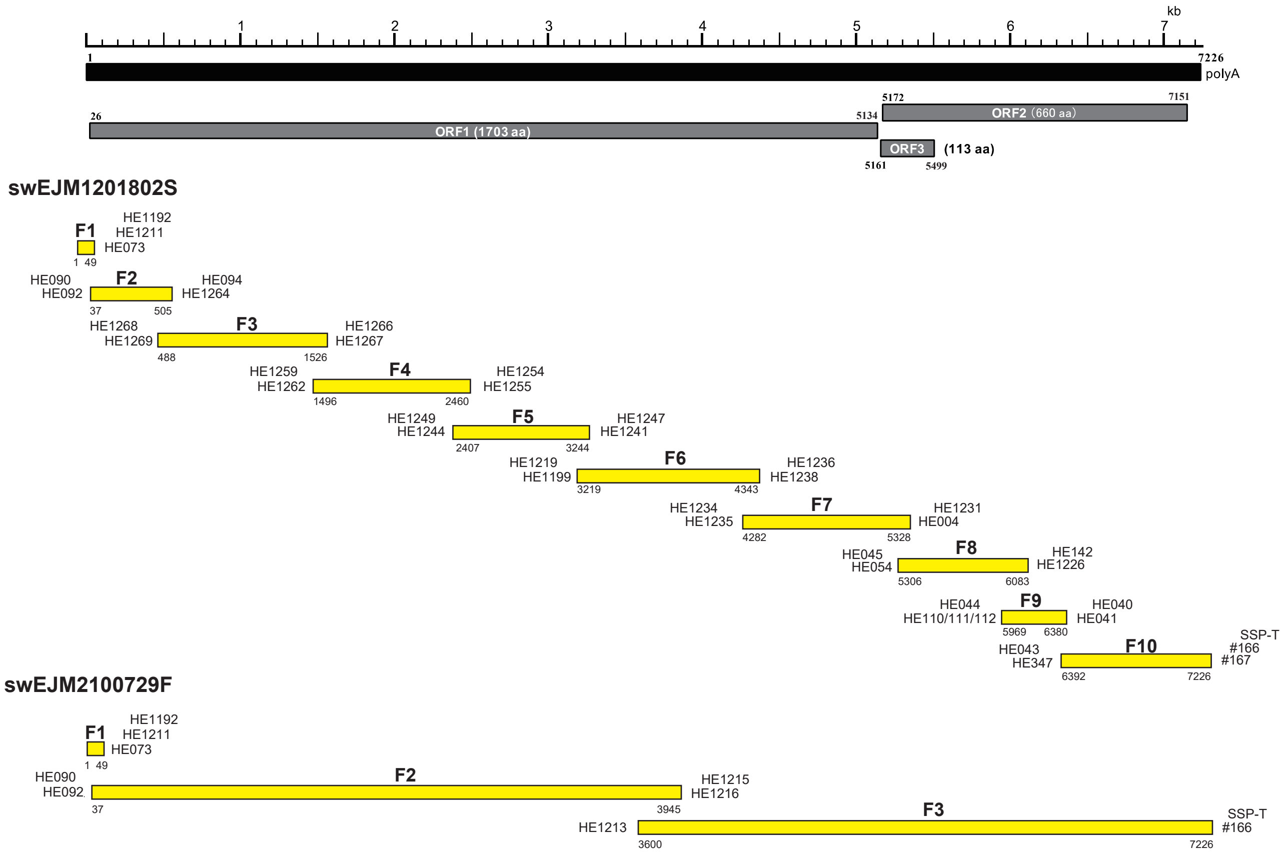

Supplementary Fig. S1. The genomic organization of HEV is illustrated at the top, and the regions amplified for nucleotide sequencing of the swEJM1201802S and swEJM2100729F genomes are depicted with shaded boxes at the bottom. The primers used for amplification of the swEJM1201802S and swEJM2100729F genomes are indicated in Supplementary Table S1 and Table S2, respectively.

Supplementary Table S1

Primers and the adaptor used for full-genome sequencing of swEJM1201802S

Region	Name	Polarity	Sequence (5' to 3')	Position	Note
1	5'Adaptor		UGGCGAUGAAUGAACACUGCGUUUGCUGGCUUUGAUGAAA	-	RNA Adaptor for 5' RLM-RACE
(5' RLM-RACE)	HE1192	-	GCATTCGCCAAGGCRGAGTTG	97 - 117	cDNA synthesis
	5'Outer Primer		TGGCGATGAATGAACACTGCG	-	1st-round PCR
	HE1211	-	GCAGCCAGAGCAGCCTGCTC	74 - 93	1st-round PCR
	RLM-RACE2		ATGAATGAACACTGCGTTTGCTGG	-	2nd-round PCR
	HE073	-	GGCAGTAGTAATGCCAGGAGC	50 - 70	2nd-round PCR
2	HE090	+	GCAGACCACRTATGTGGTCGAYGCC	1 - 25	1st-round PCR
	HE094	-	TGGCGGRMCATNGCCTCBGCRACATC	542 - 567	cDNA synthesis and 1st-round PCR
	HE092	+	TGTGGTCGAYGCCATGGAGGCCCA	13 - 36	2nd-round PCR
	HE1264	-	TCATGCAGAGAGTAAAGGGCCAC	506 - 528	2nd-round PCR
3	HE1268	+	CCTGCCGACCGTACCTACTGT	443 - 463	1st-round PCR
	HE1266	-	AAACATCAAGATATGCAGGCTCAGC	1553 - 1577	cDNA synthesis and 1st-round PCR
	HE1269	+	TGATGGATTCTCCCGCTGTGCT	466 - 487	2nd-round PCR
	HE1267	-	GGATCAACCTCAGAACCCTCATAGG	1527 - 1551	2nd-round PCR
4	HE1259	+	AGAGTGCTYGTBTTYGATGAGTCAGT	1364 - 1389	1st-round PCR
	HE1254	-	CGCTGGTAAAAGGCATGACAGAGG	2503 - 2526	cDNA synthesis and 1st-round PCR
	HE1262	+	TTYTTRGARCCAGCTGAGGGC	1475 - 1495	2nd-round PCR
	HE1255	-	CGGGTTGGAGGCATTAACCAGC	2461 - 2482	2nd-round PCR
5	HE1249	+	MCNCCCCCCGTGCGTAAGCC	2342 - 2361	1st-round PCR
	HE1247	-	CGTAACACACGGCTTGTAGTTTGG	3364 - 3387	cDNA synthesis and 1st-round PCR
	HE1244	+	CCGCCGTCTYCTTTAYACCTA	2386 - 2406	2nd-round PCR
	HE1241	-	GGGACCAAGCCAGCATGCTC	3245 - 3264	2nd-round PCR
6	HE1219	+	CGYCGTGTTGTKATTGAYGAGGC	3125 - 3147	1st-round PCR
	HE1236	-	ACACCGACTCCTCATAAGCATCAC	4362 - 4385	cDNA synthesis and 1st-round PCR
	HE1199	+	CHTCRGTCCAYCTCCTYGGTGACC	3195 - 3218	2nd-round PCR
	HE1238	-	AAGCATCACCATAAAAGATGTTAGGCG	4344 - 4370	2nd-round PCR
7	HE1234	+	TGYAAYAAGTTYACAACYGGWGAGACCAT	4208 - 4236	1st-round PCR
	HE1231	-	CCCGGATTGTGAAATGACATCG	5363 - 5384	cDNA synthesis and 1st-round PCR
	HE1235	+	CCAGGGYATATCRGCCTGGAGYAAGAC	4255 - 4281	2nd-round PCR
	HE004	-	GGGTTGGTTGGATGAATATAGGGGA	5329 - 5353	2nd-round PCR
8	HE045	+	TGCCTATGYTGCCCGCGCCAC	5212 - 5232	1st-round PCR
	HE142	-	TGGTATAYCGGGAHACACGG	6125 - 6144	cDNA synthesis and 1st-round PCR
	HE054	+	GGTGGTTTCTGGGGTGACAG	5286 - 5305	2nd-round PCR
	HE1226	-	CGGGTGTCARATTCCTAAACTCAAGCTC	6084 - 6111	2nd-round PCR
9	HE044	+	CAAGGHTGGCGYTCKGTTGAGAC	5937 - 5959	1st-round PCR
	HE040	-	CCCTTRTCCTGCTGAGCRTTCTC	6420 - 6442	cDNA synthesis and 1st-round PCR
	HE110	+	GYTCKGTTGAGACCTCYGGGGT	5947 - 5968	2nd-round PCR
	HE111	+	GYTCKGTTGAGACCACGGGYGT	5947 - 5968	2nd-round PCR
	HE112	+	GYTCKGTTGAGACCTCTGGTGT	5947 - 5968	2nd-round PCR
	HE041	-	TTMACWGTCRGCTCGCCATTGGC	6381 - 6403	2nd-round PCR
10	SSP-T	-	AAGGATCCGTCGACATCGATAATACGTTTTTTTTTTTTTTT	-	cDNA synthesis
(3' RACE)	HE043	+	ACAGAATTGATTTCGTCGGC	6324 - 6343	1st-round PCR
	#166	-	AAGGATCCGTCGACATCGAT	-	1st-round PCR
	HE347	+	GTYGTCTCRGCCAATGGCGA	6372 - 6391	2nd-round PCR
	#167	-	CCGTCGACATCGATAATACG	-	2nd-round PCR

Supplementary Table S2

Primers and the adaptor used for full-genome sequencing of swEJM2100729F

Region	Name	Polarity	Sequence (5' to 3')	Position	Note
1	5'Adaptor		UGGCGAUGAAUGAACACUGCGUUUGCUGGCUUUGAUGAAA	-	RNA Adaptor for 5' RLM-RACE
(5' RLM-RACE)	HE1192	-	GCATTCGCCAAGGCRGAGTTG	97 - 117	cDNA synthesis
	5'Outer Primer		TGGCGATGAATGAACACTGCG	-	1st-round PCR
	HE1211	-	GCAGCCAGAGCAGCCTGCTC	74 - 93	1st-round PCR
	RLM-RACE2		ATGAATGAACACTGCGTTTGCTGG	-	2nd-round PCR
	HE073	-	GGCAGTAGTAATGCCAGGAGC	50 - 70	2nd-round PCR
2	HE090	+	GCAGACCACRTATGTGGTCGAYGCC	1 - 25	1st-round PCR
	HE1215	-	CTCACAHGTRGTGGCCTGGAC	4076 - 4096	cDNA synthesis and 1st-round PCR
	HE092	+	TGTGGTCGAYGCCATGGAGGCCCA	13 - 36	2nd-round PCR
	HE1216	-	CCYTGCTCRAGCTCRGGGCA	3946 - 3965	2nd-round PCR
3	SSP-T	-	AAGGATCCGTCGACATCGATAATACGTTTTTTTTTTTTTTT	-	cDNA synthesis
(3' RACE)	HE1213	+	YACCCGYCAYACAGAGAAGTGC	3577 - 3599	1st-round PCR
	#166	-	AAGGATCCGTCGACATCGAT	-	1st-round PCR
